# Plasminogen Activator Inhibitor-Type I Gene Deficient Mice Show Reduced Influx of Neutrophils in Ventilator-Induced Lung Injury

**DOI:** 10.1155/2011/217896

**Published:** 2011-07-14

**Authors:** Esther K. Wolthuis, Alexander P. J. Vlaar, Jorrit-Jan H. Hofstra, Joris J. T. H. Roelofs, Vivian de Waard, Nicole P. Juffermans, Marcus J. Schultz

**Affiliations:** ^1^Department of Anesthesiology, Academic Medical Center, University of Amsterdam, 1105 AZ Amsterdam, The Netherlands; ^2^Laboratory of Experimental Intensive Care and Anesthesiology (LEICA), Academic Medical Center, University of Amsterdam, 1105 AZ Amsterdam, The Netherlands; ^3^Department of Internal Medicine, Academic Medical Center, University of Amsterdam, 1105 AZ Amsterdam, The Netherlands; ^4^Department of Pathology, Academic Medical Center, University of Amsterdam, 1105 AZ Amsterdam, The Netherlands; ^5^Department of Medical Biochemistry, Academic Medical Center, University of Amsterdam, 1105 AZ Amsterdam, The Netherlands; ^6^Department of Intensive Care Medicine, Academic Medical Center, University of Amsterdam, 1105 AZ Amsterdam, The Netherlands; ^7^HERMES Critical Care Group, Amsterdam, The Netherlands

## Abstract

Ventilator-induced lung injury (VILI) is associated with inhibition of the fibrinolytic system secondary to increased production of plasminogen activator inhibitor- (PAI-)1. To determine the role of PAI-1 on pulmonary coagulopathy and inflammation during mechanical ventilation, PAI-1 gene-deficient mice and their wild-type littermates were anesthetized (control), or anesthetized, tracheotomized and subsequently ventilated for 5 hours with either low tidal volumes (LV_T_) or high tidal volumes (HV_T_). VILI was assessed by pulmonary coagulopathy, lung wet-to-dry ratios, total protein level in bronchoalveolar lavage fluid, neutrophil influx, histopathology, and pulmonary and plasma cytokine levels. Ventilation resulted in pulmonary coagulopathy and inflammation, with more injury following ventilation with HV_T_ as compared to LV_T_. In PAI-1 gene-deficient mice, the influx of neutrophils in the pulmonary compartment was attenuated, while increased levels of pulmonary cytokines were found. Other endpoints of VILI were not different between PAI-1 gene-deficient and wild-type mice. These data indicate that a defect fibrinolytic response attenuates recruitment of neutrophils in VILI.

## 1. Introduction

Next to alveolar tissue factor-mediated thrombin generation and impaired activity of endogenous inhibitors of coagulation, depressed alveolar fibrinolysis is a typical feature of pneumonia and acute lung injury (ALI) [1, 2]. Early mediators of fibrinolysis are plasminogen activators (PAs), which activate plasminogen into plasmin, a potent protease that degrades fibrin into fibrin degradation products. PAs are controlled by specific inhibitors, of which plasminogen activator inhibitor type 1 (PAI-1) is considered most important, inactivating both urokinase-type PA (uPA) and tissue-type PA (tPA). The pulmonary compartment is an important site of PAI-1 production and activity. Elevated bronchoalveolar lavage fluid (BALF) PAI-1 levels correlate with increased morbidity and mortality of patients suffering from pneumonia [3, 4] or ALI [5]. A potential role of PAI-1 in the pathogenesis of lung inflammation is further suggested by its upregulation in various experimental models of ALI [6, 7] and by the finding that mice genetically deficient in PAI-1 fail to accumulate alveolar fibrin and die less early in response to hyperoxia [8].

There is compelling evidence from observations in humans and in experimental models that neutrophils are primary perpetrators of inflammatory injury to the lung. Indeed, neutrophil influx into the alveolar space correlates with lung injury as manifest, by an increase in permeability of the alveolocapillary membrane [9]. In addition, in models of ALI neutrophil depletion is protective [10, 11]. During and after the translocation of neutrophils from the vasculature into the airspaces, excessive and/or prolonged activation leads to extracellular release of soluble mediators, including elastase, matrix metalloproteinases, defensins, and oxidants [12]. These mediators all induce epithelial cell apoptosis and sloughing, resulting in enhanced permeability of the alveolocapillary membrane, which allows for extravasation of plasma into the alveolar spaces leading to compromised gas exchange and diminished lung compliance.

Several studies indicate that the fibrinolytic system may influence pulmonary inflammation. First, mice lacking the receptor for uPA, which is expressed on different inflammatory cells, have an impaired leukocyte migration [13, 14]. Second, PAI-1 may affect neutrophil trafficking in several ways. Indeed, PAI-1 is not only an inhibitor of uPA in the lung but can also interfere with cell adhesion in a more direct way [15, 16]. Additional evidence supporting a role for PAI-1 in cell migration comes from tumor cell biology which shows that high expression of PAI-1 is predictive of more aggressive local invasion and metastasis and is a poor prognostic marker [17–19].

Mechanical ventilation (MV) with potentially injurious ventilator settings (high tidal volumes (V_T_) and no positive end-expiratory pressure (PEEP)) induces upregulation of PAI-1 in the pulmonary compartment in patients without preexisting lung injury [20]. These findings are confirmed in experimental models of ventilator-induced lung injury (VILI) in which high V_T_ attenuate alveolar fibrinolytic activity [21, 22]. This appears to be caused, at least in part, by increased local production of PAI-1. Infiltration of neutrophils concomitant with the development of physiological signs of lung injury is a characteristic feature of VILI [23–25]. The direct relation between local PAI-1 activity and neutrophil infiltration during VILI, however, has never been elucidated. Therefore, the main objective of this study was to determine the role of endogenous PAI-1 in alveolar coagulopathy and pulmonary inflammation, in particular neutrophil infiltration, in response to mechanical ventilation. For this, we used an MV model in mice comparing low and high V_T_, using PAI-1 gene-deficient (PAI-1^−/−^) mice.

## 2. Materials and Methods

The study was approved by the Animal Care and Use Committee of the Academic Medical Center of the University of Amsterdam, Amsterdam, The Netherlands. Animal procedures were carried out in compliance with Institutional Standards for Human Care and Use of Laboratory Animals.

### 2.1. Animals

PAI-1 gene-deficient (PAI-1^−/−^) mice on a C57Bl/6 genetic background (*n* = 36) and normal C57Bl/6 wild-type (Wt) mice (*n* = 36) were obtained from the Jackson Laboratory (Bar Harbor, ME). PAI-1^−/−^ mice exhibit normal fertility, viability, tissue histology, and development and show neither evidence of macroscopic or microscopic histological abnormalities nor hemorrhage [26]. Female mice with weights ranging from 18 to 22 grams were used in all experiments. Nonventilated anesthetized mice served as controls (*n* = 12 for PAI-1^−/−^ mice and Wt mice). Tracheotomized mice were connected to a ventilator and ventilated with 2 different MV strategies (*n* = 12 for PAI-1^−/−^ mice and Wt mice for every different MV strategy).

### 2.2. Instrumentation and Anesthesia

Anesthesia consisted of intraperitoneal injection of a mix of ketamine (Eurovet Animal Health BV, Bladel, the Netherlands), medetomidine (Pfizer Animal Health BV, Capelle a/d IJssel, The Netherlands), and atropine (Pharmachemie, Haarlem, the Netherlands) (KMA). Induction anesthesia was achieved with intraperitoneal injection of KMA “induction” mix: 7.5 *μ*L per gram of body weight of 1.26 mL 100 mg/mL ketamine, 0.2 mL 1 mg/mL medetomidine, and 1 mL 0.5 mg/mL atropine in 5 mL normal saline. Maintenance anesthesia was achieved with hourly intraperitoneal injection of 10 *μ*L per gram body weight of KMA “maintenance” mix, consisting of 0.72 mL 100 mg/mL ketamine, 0.08 mL 1 mg/mL medetomidine, and 0.3 mL 0.5 mg/mL atropine, in 20 mL normal saline. Maintenance mix was administered via an intraperitoneal catheter (PE 10 tubing, BD, Breda, The Netherlands) every hour.

### 2.3. Mechanical Ventilation

A Y-tube connector, 1.0 mm outer diameter and 0.6 mm inner diameter (VBM Medizintechnik GmbH, Sulz am Neckar, Germany), was surgically inserted into the trachea under general anesthesia. Mice were placed in a supine position and connected to a ventilator (Servo 900C, Siemens, Sweden). Mice were ventilated with either an inspiratory pressure of 10 cmH_2_O (resulting in lung-protective V_T_ ~ 7.5 mL/kg; low V_T_, LV_T_) or an inspiratory pressure of 18 cmH_2_O (resulting in injurious V_T_ ~ 15 mL/kg; high V_T_, HV_T_). Respiratory rate was set at 120 breaths/min and 70 breaths/min with LV_T_ and HV_T_, respectively. PEEP was set at 2 cmH_2_O with both MV strategies. Respiratory settings resulted in normal PaCO_2_ values after 5 h of MV with both strategies [27]. The fraction of inspired oxygen was kept at 0.5. The inspiration to expiration ratio was kept at 1 : 1 throughout the experiment. A sigh (sustained inflation with 30 cmH_2_O) for 5 breaths was performed every 30 minutes. Mice received intraperitoneal fluid boluses (normal saline 1 hour before start of anesthesia and initiation of MV, followed by 0.2 mL sodium bicarbonate (200 mmol/L NaHCO_3_) administered via the intraperitoneal catheter every 30 minutes until the end of MV).

### 2.4. Monitoring

Throughout the experiments rectal temperature was monitored and maintained between 36.0 and 37.5°C using a warming path. Systolic blood pressure and heart rate were noninvasively monitored using a murine tail-cuff system (ADInstruments, Spenbach, Germany). Blood pressure and pulse were measured directly after start of MV, after 2.5 and 5 hours of MV. The data were recorded on a data acquisition system (PowerLab/4SP, ADInstruments). Systolic blood pressure and heart rate were averaged from 3 consecutive measurements. V_T_ was checked hourly with a plethysmograph system. A minimum of 5 consecutive breaths was selected for analysis of the digitized V_T_ signals.

### 2.5. Study Groups

Nonventilated mice received half the dose of induction anesthesia, were spontaneously breathing, and sacrificed after 5 hours (control mice). LV_T_ mice and HV_T_-mice were mechanically ventilated for 5 hours and then sacrificed. Half of these mice were sacrificed for blood sampling, drawn from the vena cava inferior into a sterile syringe, transferred to EDTA-coated tubes, and immediately placed on ice. Subsequently, bronchoalveolar lavage fluid (BALF) was obtained from the right lung; the left lung was used to measure wet-to-dry ratios (W/D). The other half of these mice were sacrificed for blood gas analysis, and blood was sampled from the carotid artery. The lungs of these mice were used for homogenate (right lung) and histopathology (left lung).

### 2.6. Blood Gas Analysis and Blood Sample Handling

For blood gas analysis, blood was immediately analyzed in a Rapidlab 865 blood gas analyzer (Bayer, Mijdrecht, The Netherlands). The other blood samples were centrifuged at 3000 rpm at 4°C for 10 min, and the supernatants were aliquoted and frozen at −80°C until assayed.

### 2.7. Lung Wet-to-Dry Ratios (W/D)

For W/D the left lung was weighed and subsequently dried for 3 days in an oven at 65°C. The ratio of wet weight to dry weight represents tissue edema.

### 2.8. Bronchoalveolar Lavage

BALF was obtained from the right lung by instilling three times 0.5 mL aliquots of saline by a 22-gauge Abbocath-T catheter (Abbott, Sligo, Ireland) into the trachea. Approximately, 1.1 mL of lavage fluid was retrieved per mouse, and cell counts were determined using a hemacytometer (Beckman Coulter, Fullerton, Calif). Subsequently, differential counts were done on cytospin preparations stained with a modified Giemsa stain, Diff-Quick (Dade Behring AG, Düdingen, Switzerland). Supernatant was stored at −80°C for total protein level, thrombin-antithrombin complexes, and PAI-1 levels.

### 2.9. Lung Homogenates

During sacrificing the right lung was removed and snap frozen in liquid nitrogen. Lung homogenates were prepared as described before [28]. In short, frozen specimens were weighed and suspended in 4 volumes of sterile isotonic saline and subsequently lysed in 1 volume of lysis buffer (150 mM NaCl, 15 mM Tris [tris(hydroxymethyl)aminomethane], 1 mM MgCl.H_2_O, 1 mM CaCl_2_, 1% Triton X-100, 100 *μ*g/mL pepstatin A, leupeptin and aprotinin, pH 7.4) and incubated at 4°C for 30 min. Homogenates were spun at 3400 rpm at 4°C for 15 minutes after which the supernatants were stored at −80°C until assayed.

### 2.10. Histopathology

For histopathology lungs were fixed in 4% formalin and embedded in paraffin. 4 *μ*m sections were stained with hematoxylin-eosin (H&E), and analyzed by a pathologist who was blinded for group identity. To score lung injury we used a modified VILI histology scoring system as previously described [23]. In short, four pathologic parameters were scored on a scale of 0–4: (a) alveolar congestion, (b) hemorrhage, (c) leukocyte infiltration, and (d) thickness of alveolar wall/hyaline membranes. A score of 0 represents normal lungs; 1, mild, <25% lung involvement; 2, moderate, 25−50% lung involvement; 3, severe, 50–75% lung involvement, and 4, very severe, >75% lung involvement. The total histology score was expressed as the sum of the score for all parameters.

### 2.11. PAI-1 *In Situ* Hybridization

PAI-1 *in situ* hybridization was performed as described before [29]. In short, 7-*μ*m-thick paraffin sections of lung tissue were mounted on SuperFrost Plus glass slides (Menzel-Gläser, Braunschweig, Germany) and subjected to *in situ* hybridization using radiolabeled [^35^S]-UTP (Amersham, Arlington Heights, Ill, USA) mouse PAI-1-specific riboprobes. *In situ* hybridization was executed by standard procedures. *In situ* sections were covered with autoradiography emulsion (Ilford G5 emulsion 1 : 1 diluted with 2% glycerol). Slides were developed in Kodak D19 after an exposure of 5 weeks, fixed in Kodak UNIFIX, and counterstained with hematoxylin and eosin. Four sections per specimen were used to confirm positive PAI-1 *in situ* hybridization signal.

### 2.12. Assays

Total protein levels in BALF were determined using a Bradford Protein Assay Kit (OZ Biosciences, Marseille, France) according to manufacturers' instructions with bovine serum albumin as standard. Cytokine and chemokine levels in lung homogenates were measured by enzyme-linked immunosorbent assay (ELISA) according to the manufacturer's instructions. Tumor necrosis factor *α* (TNF), interleukin- (IL-) 6, macrophage inflammatory protein- (MIP-) 2, and keratinocyte-derived chemokine (KC) assays were all obtained from R&D Systems (Abingdon, UK).

Thrombin-antithrombin complex levels in BALF were measured with a mouse-specific ELISA as previously described [30]. PAI-1 was measured with ELISA (Kordia, Leiden, The Netherlands). To measure plasmin activity, levels of fibrin degradation products, the split product cleaved off from cross-linked fibrin by a direct action of plasmin, were measured by means of ELISA (Asserachrom D-dimer, Roche, Woerden, The Netherlands).

### 2.13. Statistical Analysis

All data in the results are expressed as individual data in the figures. To detect differences between mechanical ventilation groups, the Dunnett method, in conjunction with two-way analysis of variance, was used. For differences between PAI-1^−/−^ and Wt mice, post hoc analysis with Mann Whitney *U* test was done. A *P* value of <0.05 was considered significant. All statistical analyses were carried out using SPSS 12.0.2 (SPSS, Chicago, Ill).

## 3. Results

All instrumented animals survived 5 hours of MV after which they were sacrificed. Hemodynamic monitoring demonstrated stable conditions throughout the experiment; systolic blood pressure and heart rate remained stable in all animals for the complete duration of MV. Blood gas analyses were not different between both ventilation groups and between PAI-1^−/−^ and Wt mice.

### 3.1. Production of PAI-1 with Low and High *V*_*T*_

**Figure 1:**
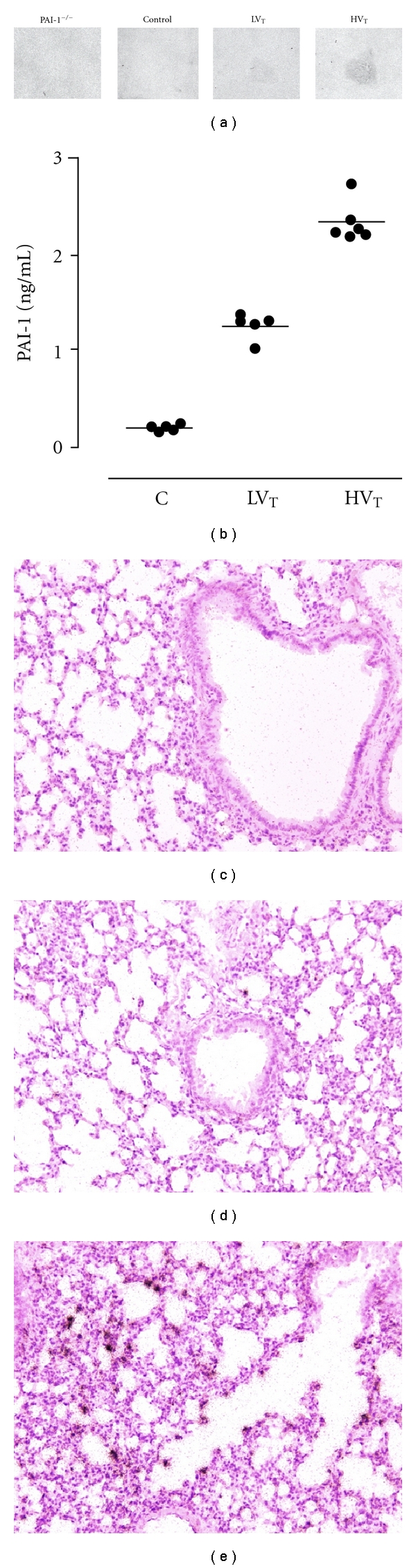
(a) Plasminogen activator inhibitor- (PAI-) 1 mRNA in lung. PAI-1 mRNA as determined by radioactive *in situ* hybridization (autoradiographic film). (b) PAI-1 levels in bronchoalveolar lavage fluid in anesthetized nonventilated control (c) mice, mice ventilated with low tidal volumes (LV_T_) and high V_T_ (HV_T_). Levels of PAI-1 in PAI-1^−/−^ mice were undetectable. Data are represented as individual data with a median. *In situ* hybridization for murine PAI-1 mRNA was performed on paraffin slides of nonventilated control Wt mice (c), Wt mice ventilated with LV_T_ (d) and HV_T_ (e) for 5 hours. Positive signal in black. Original magnification 100x.

To confirm PAI-1 production in our model, we measured PAI-1 levels in BALF. MV increased the concentrations of PAI-1 after 5 hours of MV as compared to control mice (*P* < 0.001 for LV_T_ mice and HV_T_ mice; Figure 1). To obtain insight into the cellular source of PAI-1 in the normal and injured lung, we performed *in situ* hybridization. In normal lung, a faint signal for PAI-1 mRNA transcripts was detected in endothelial cells as well as in bronchiolar and alveolar epithelium (Figure 1). After 5 hours of MV, a strong expression of PAI-1 mRNA was observed, predominantly in vessels showing endothelial injury, in inflamed bronchi, in areas of inflammatory infiltrates, and in areas of pleuritis. 

### 3.2. Pulmonary Coagulopathy

**Figure 2:**
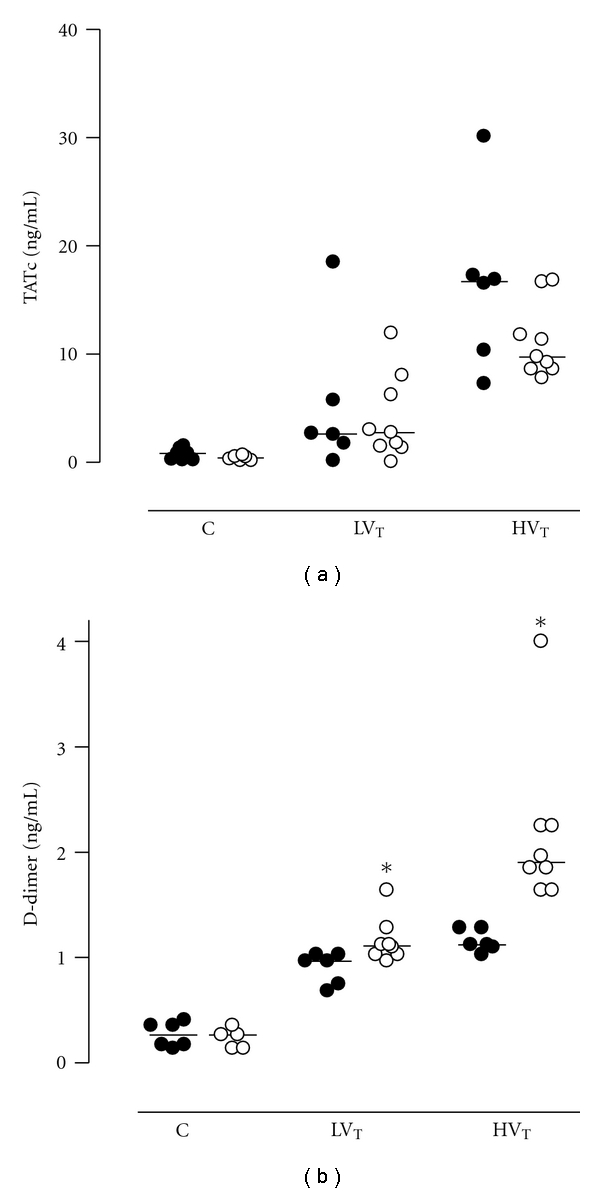
Thrombin-antithrombin complexes (TATcs) levels in bronchoalveolar lavage fluid and levels of fibrin degradation products in lung homogenate in anesthetized nonventilated control (C) mice, mice ventilated with LV_T_ or HV_T_. Mice were either PAI-1 deficient (PAI-1^−/−^, open symbols) or wild type (Wt, closed symbols). Data are represented as individual data with a median. *, indicates statistical significant difference compared to Wt.

BALF TATc levels were higher in both ventilation groups as compared to control mice (*P* = 0.049 for LV_T_ mice and *P* < 0.001 for HV_T_ mice), with higher levels in HV_T_ mice (*P* < 0.001 versus LV_T_) mice; Figure 2). There was no significant difference between PAI-1^−/−^ mice and Wt mice regarding activation of coagulation in the pulmonary compartment. Levels of fibrin degradation products were higher in both ventilated groups as compared to control (*P* < 0.001 for LV_T_ mice and HV_T_ mice), with higher levels in HV_T_ mice (*P* < 0.001 versus LV_T_ mice; Figure 2). In PAI-1^−/−^ mice fibrinolysis was enhanced during MV, as reflected by a stronger increase in levels of fibrin degradation products (Figure 2). 

### 3.3. Lung Injury

**Figure 3:**
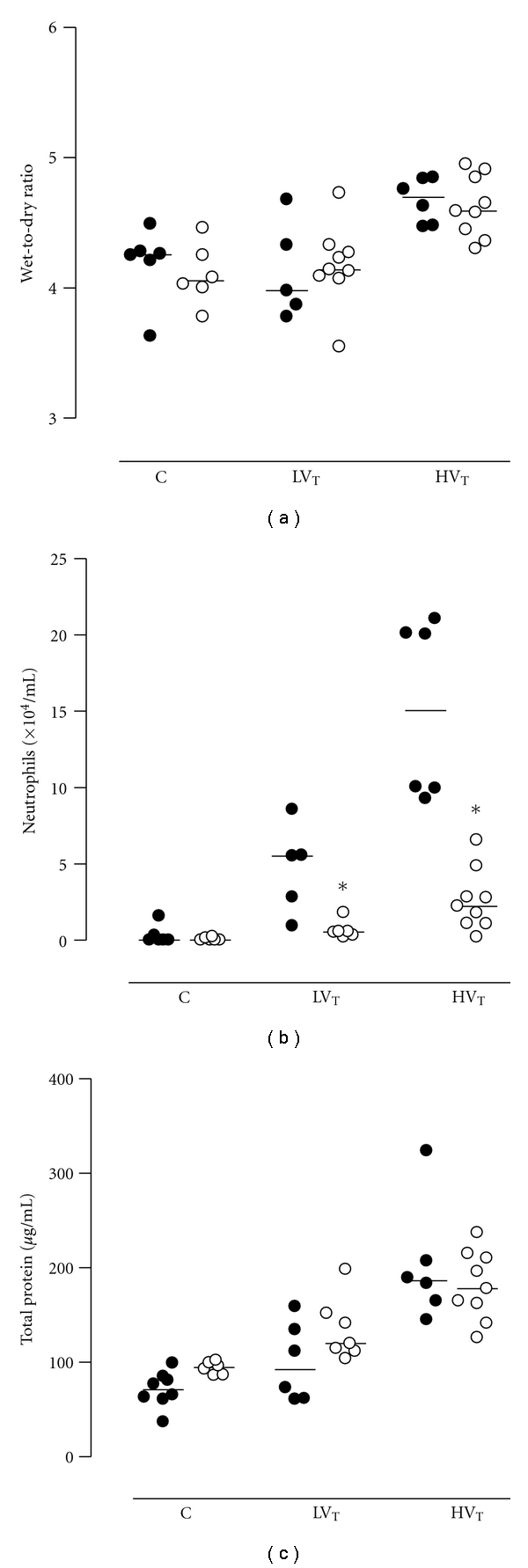
Wet-to-dry ratios (W/D) of the left lung, total protein level in BALF, and number of neutrophils in BALF in anesthetized nonventilated control (C) mice, mice ventilated with LV_T_ or HV_T_. Mice were either PAI-1 deficient (PAI-1^−/−^, open symbols) or wild type (Wt, closed symbols). Data are represented as individual data with a median. * indicates statistical significant difference compared to Wt.

Lung W/D was significantly higher in HV_T_ mice as compared to LV_T_ mice and controls (*P* < 0.001; Figure 3). In accordance, total BALF protein levels were higher in both ventilation groups (*P* = 0.049 for LV_T_ mice and *P* < 0.001 for HV_T_ mice), with higher levels in HV_T_ mice (*P* < 0.001; Figure 3). The number of neutrophils in BALF was higher in both HV_T_ and LV_T_ mice as compared to control (*P* < 0.001 and *P* = 0.047, resp.). The difference between HV_T_ mice and LV_T_ mice was significant (*P* < 0.001; Figure 3). 

No differences were seen between PAI-1^−/−^ mice and Wt mice regarding W/D and total protein in BALF (Figure 3). However, in PAI-1^−/−^ mice, there was less neutrophil influx in both ventilated groups as compared to Wt mice (*P* = 0.004 for LV_T_ mice and *P* = 0.002 for HV_T_ mice; Figure 3).

Histopathological changes were minor. The VILI score in HV_T_ mice was significantly higher as compared to control mice (*P* = 0.026). No difference in VILI score was observed between LV_T_ mice and HV_T_ mice. Finally, histopathological lung injury scores were not different between PAI-1^−/−^mice and Wt mice.

### 3.4. Pulmonary and Systemic Inflammation

**Figure 4:**
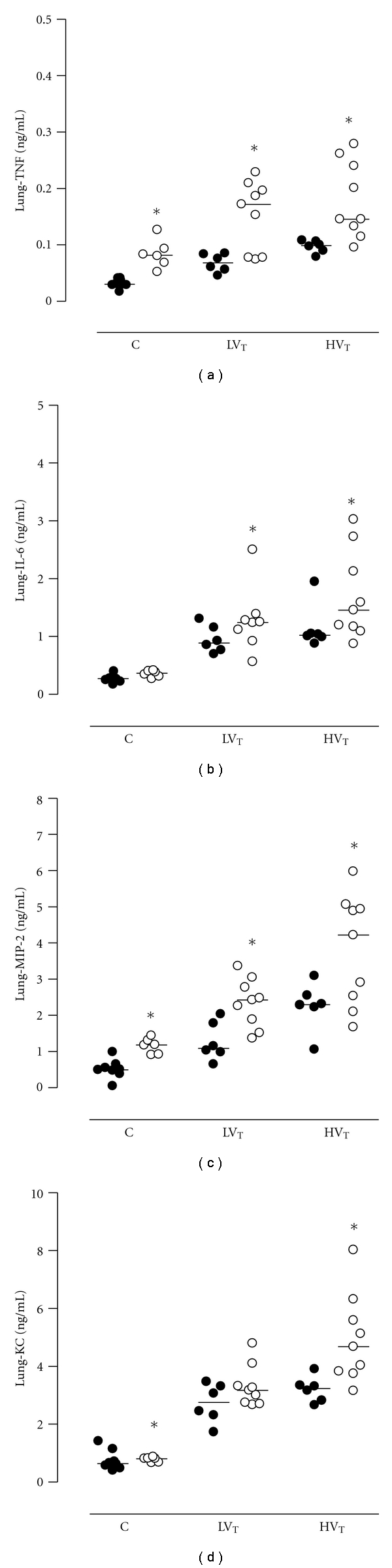
Pulmonary levels of tumor necrosis factor *α* (TNF), interleukin- (IL-) 6, macrophage inflammatory protein- (MIP-) 2, and keratinocyte-derived chemokine (KC) in lung tissue homogenate in anesthetized nonventilated control (C) mice, mice ventilated with LV_T_ or HV_T_. Mice were either PAI-1 deficient (PAI-1^−/−^, open symbols) or wild type (Wt, closed symbols). Data are represented as individual data with a median. * indicates statistical significant difference compared to Wt.

Ventilated mice demonstrated higher pulmonary levels of TNF and IL-6 as compared to control mice (*P* < 0.001 for LV_T_ mice and HV_T_ mice). For both cytokines there was no significant difference between LV_T_ and HV_T_ mice, however (Figure 4). Ventilated mice also demonstrated higher pulmonary levels of MIP-2 and KC as compared to control (*P* < 0.001 for LV_T_ and HV_T_ mice), with higher levels of MIP-2 and KC in HV_T_ mice (*P* < 0.001 versus LV_T_ mice; Figure 4). PAI-1^−/−^ mice demonstrated higher pulmonary TNF and IL-6 levels in nonventilated control mice (*P* = 0.002 and *P* = 0.009, resp.; Figure 4). For both ventilated groups pulmonary TNF and IL-6 levels were significantly higher in PAI-1^−/−^ mice as compared to Wt mice. Pulmonary levels of MIP-2 and KC were elevated in all PAI-1^−/−^ mice, except for KC levels in the LV_T_ group (*P* = 0.13).

**Figure 5:**
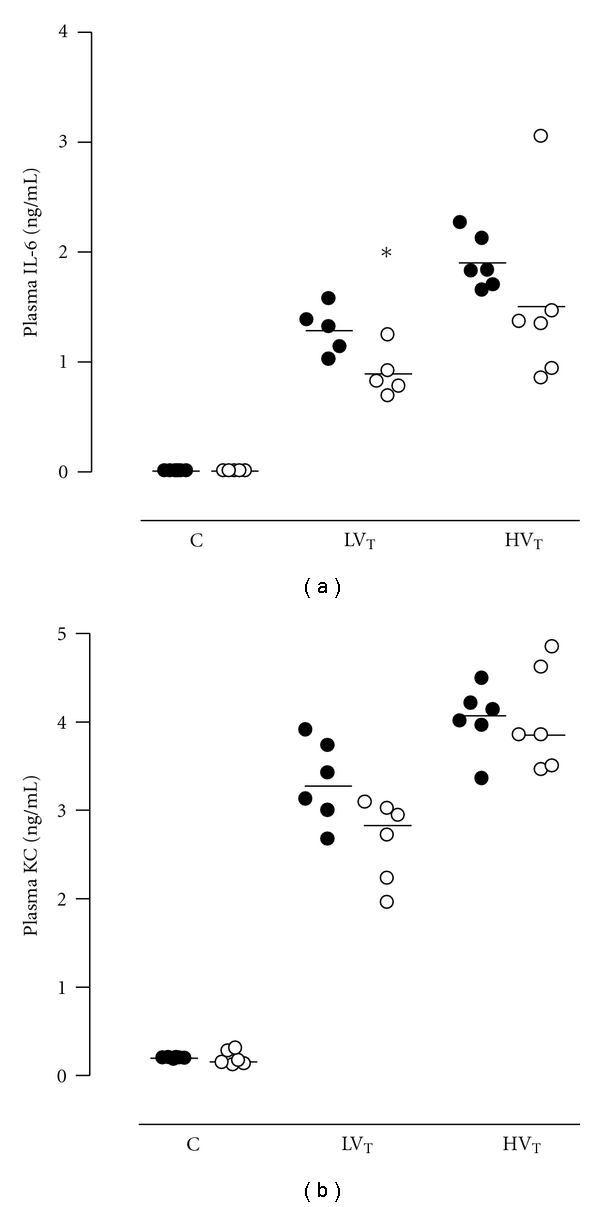
Systemic levels of interleukin- (IL-) 6, and keratinocyte-derived chemokine (KC) in plasma in anesthetized nonventilated control (C) mice, mice ventilated with LV_T_ or HV_T_. Mice were either PAI-1 deficient (PAI-1^−/−^, open symbols) or wild type (Wt, closed symbols). Data are represented as individual data with a median. * indicates statistical significant difference compared to Wt.

Plasma levels of IL-6 and KC were elevated in both ventilation groups as compared to control (*P* < 0.001 for both LV_T_ and HV_T_ mice), with higher levels in HV_T_ mice (*P* < 0.001; Figure 5). Plasma levels of IL-6 were lower in PAI-1^−/−^ mice in the LV_T_ group as compared to Wt mice (*P* = 0.009; Figure 5). HV_T_ mice demonstrated a trend for lower IL-6 levels in PAI-1^−/−^ mice (*P* = 0.065). For plasma levels of KC, no differences were observed between PAI-1^−/−^mice and Wt mice. 

## 4. Discussion

There is mounting evidence indicating that PAI-1 plays a potent role in the procoagulant response with various forms of lung injury. The results from the present study suggest that PAI-1 is responsible, at least in part, for neutrophil influx into the alveolar space with MV-induced lung injury, since PAI-1^−/−^ mice show less neutrophil influx as compared to Wt mice. On the other hand, PAI-1^−/−^ mice show increased pulmonary levels of inflammatory mediators, even in the control, nonventilated mice. Plasma levels of IL-6 were lower in PAI-1^−/−^ mice as compared to Wt mice. Notably, PAI-1 deficiency did not result in changes of other endpoints of lung injury, that is, gas exchange, W/D, BALF protein levels, and histopathology.

PAI-1 is first of all a strong inhibitor of fibrinolysis. In line, PAI-1^−/−^ mice have an accelerated spontaneous whole blood clot lysis [26]. We here show that PAI-1 is upregulated during MV, with higher levels of PAI expression during high tidal volume ventilation. In addition D-dimer concentrations, as a reflection of fibrinolysis, are elevated during MV. In PAI-1 deficient mice fibrinolysis is enhanced. Besides its role as a regulator of haemostasis by regulating fibrinolytic activity, PAI-1 plays a role in many other (patho)physiological processes, including wound healing, atherosclerosis, tumor angiogenesis, pulmonary fibrosis, and kidney disease [18, 19, 25, 29, 31, 32]. In addition PAI-1 acts as an acute phase protein during sepsis [33], and plasma PAI-1 levels rise markedly during disease states often associated with a sterile acute phase response including trauma and surgery [34]. More recently, PAI-1 has been shown to be critically involved in the regulation of cell migration. PAI-1 can inhibit cell bound uPA, resulting in reduced pericellular proteolysis and a subsequent decrease in cell migration. On the contrary, deadhesive action of PAI-1 by inactivation of the cell-integrin-extracellular matrix interaction may result in an increase of cell mobility as well. So PAI-1 seems to exert both promoting and inhibitory effects on cell migration.

The direct relation between local PAI-1 activity and neutrophil infiltration during VILI was recently described by Liu et al. [35]. They ventilated mice with either 6 or 30 mL/kg and added hyperoxia. High-tidal-volume ventilation of the mouse induced the microvascular permeability, neutrophil influx, TNF-*α*, and PAI-1 production. The addition of hyperoxia augmented this deleterious effect on injurious mechanical ventilation and was dependent, at least in part, on the NF-*κ*B pathway. Inhibition of NF-K*β* reduced TNF-*α*, PAI-1, and MPO activity in mice ventilated with high V_T_ and hyperoxia. Increases of PAI-1 on airway epithelium was reduced after pretreatment with anti-TNF-*α* antibody which implied that PAI-1 expression was partly induced by TNF-*α*. Several studies with infectious agents have shown a decreased pulmonary neutrophil influx in PAI-1^−/−^ mice [28, 29]. Neutrophils are thought to play a predominant role during the initiation of VILI [24, 36]. In our well-established MV model [27, 37] we used physiological and clinically relevant V_T_, which enhances translation of results into clinical practice. Our results of attenuated neutrophil influx in PAI-1^−/−^ mice are in line with several other reports. First, in a *Klebsiella pneumoniae *pneumonia model in mice, PAI-1^−/−^ mice demonstrated less infiltrating neutrophils in their lungs [28]. A diminished influx of neutrophils into the BALF of PAI-1^−/−^ mice has also been found after local pulmonary LPS exposure [7]. Also in a mice model of acute pyelonephritis lower neutrophil influx was observed in PAI-1^−/−^ mice as compared to Wt mice [29]. Our findings are also in line with the results obtained in a model of bleomycin-induced lung injury, in which PAI-1 gene deficiency protected against inflammation-induced lung damage and overexpression of PAI-1 enhanced the accumulation of neutrophils in the lung [38]. Furthermore, in an antigen-induced arthritis model PAI-1^−/−^ mice showed significantly reduced joint inflammation [39]. In a model of glomerulonephritis PAI-1 deficiency reduced the number of infiltrating neutrophils in the glomeruli, while mice overexpressing PAI-1 showed a profound increase in neutrophil infiltration [40]. Our data extend these findings by showing that neutrophil infiltration is attenuated in PAI-1^−/−^ mice in a model of VILI. 

Besides less neutrophil influx in PAI-1^−/−^ mice, we found increased pulmonary levels of inflammatory cytokines, even in the control, nonventilated mice. One explanation might be the genetic background of transgenic animals, which can influence different responses. The responses of transgenic animals to a challenge thus reflect the dysfunction (or alteration of the function) of the gene of interest plus compensatory mechanisms. It can also be hypothesized that the cytokine levels in Wt mice peaked earlier and that mice deficient for PAI-1 show a slower pulmonary cytokine response. In a recently published paper of Hegeman et al. they show that the cytokine peak in the lung is after two hours of MV in healthy mice ventilated with a peak inspiratory pressure of 20 cmH_2_O and 0 cmH_2_O PEEP [41]. In our study we only measured cytokine levels after 5 hours of MV. On the other hand the systemic cytokines in PAI-1^−/−^ are not increased as compared to Wt mice. IL-6 levels are even decreased in PAI-1^−/−^ mice. These cytokines will eventually be detrimental in causing multisystem organ failure [42, 43].

Our study has several limitations, though. First, only less neutrophil influx was observed for PAI-1^−/−^ mice as compared to Wt mice, with no differences in other endpoints of VILI, such as W/D or protein leakage in BALF. Because we used less high V_T_ as compared to other models of VILI, the challenge in our setting may be too mild to observe these differences. Second, our nonventilated control animals were not sham operated, did not receive fluid resuscitation, and were breathing room air as opposed to our ventilated animals. One last shortcoming of our study lies in the use of genetically modified animals. The genetic background of transgenic animals can influence different responses. Indeed, when we knock out or knock down a gene from the genome, it may trigger compensatory mechanisms. The responses of transgenic animals to a challenge thus reflect the dysfunction (or alteration of the function) of the gene of interest plus compensatory mechanisms. The increased level of pulmonary cytokines in PAI-1^−/−^ mice, even in the control, nonventilated mice, could be due to this phenomenon. Therefore, the lack of difference for hard endpoints of VILI (like W/D and protein leakage in BALF) between PAI-1^−/−^ mice and Wt mice cannot exclude the possibility of using anti-PAI-1 therapies for VILI. Additional studies are needed to test this hypothesis. 

Recently angiotensin-converting enzyme activity was shown to be an additional pathway regulating PAI-1 expression [44]. Administration of exogenous PAI-1 reversed the inhibitory effect of enalapril on neutrophil influx to the lung after exposure to LPS. These data strongly suggest that the role of PAI-1 in inflammatory cell migration is often stimulatory. Neutrophil recruitment into the lung is believed to be a critical step in the pathogenesis of ALI and results from the release of a milieu of cytokines and chemokines that precedes ALI [45]. Recently, it was shown that a neutrophil elastase inhibitor could attenuate VILI in mice. Indeed, mice that received the neutrophil elastase inhibitor showed complete inhibition of neutrophil elastase and myeloperoxidase activities, attenuation of neutrophil accumulation and lung water content [46]. In another VILI model pretreatment with a matrix metalloproteinase-9 inhibitor resulted in a decreased degree of VILI, as reflected by W/D and pathology score, and decreased neutrophil infiltration [47]. Matrix metalloproteinase-9, which is produced by neutrophils and other inflammatory cells, plays an important role in neutrophil migration.

Our results indicate that PAI-1 deficiency is associated with less neutrophil recruitment into the lung. Also, PAI-1 seems to be a very sensitive biomarker for detection of ventilator-induced lung injury in our MV model as opposed to other biomarkers measured. Elevated pulmonary PAI-1 levels correlate with poor outcome in patients with pneumonia [3, 4, 48]. Similarly, in ALI/ARDS elevated plasma levels of PAI-1 are associated with increased morbidity and mortality [5].

The value of increased pulmonary cytokines in PAI-1^−/−^ mice as compared to Wt mice is difficult, because they are already increased in nonventilated control mice. More important are the lower plasma IL-6 levels in PAI-1^−/−^mice as compared to Wt mice, since IL-6 can lead to multiorgan dysfunction syndrome [43].

## 5. Conclusions

PAI-1 gene deficiency attenuates recruitment of neutrophils into the alveolar space during mechanical ventilation. This suggests PAI-1 to play a stimulatory role of cell migration into the alveoli, which is independent of local production of chemokines. PAI-1 gene deficiency causes lower plasma IL-6 levels even in the presence of increased pulmonary cytokines. PAI-1 seems to be a very sensitive biomarker for the detection of ventilator-induced lung injury and could be used for ICU patients at risk for developing ventilator-associated lung injury.

Further studies are needed before inhibition of PAI-1 is to be tested in clinical trials of patients with or at risk for ventilator-associated lung injury.
